# Supplementary material for: Palaeogeographic regulation of glacial events during the Cretaceous supergreenhouse
Source: Nat Commun. 2016 Sep 21;7:12771. doi: 10.1038/ncomms12771 (PMC5036002; doi:10.1038/ncomms12771)
Supplement: Supplementary Information — Supplementary Figures 1-13 and Supplementary Table 1 [file ncomms12771-s1.pdf]

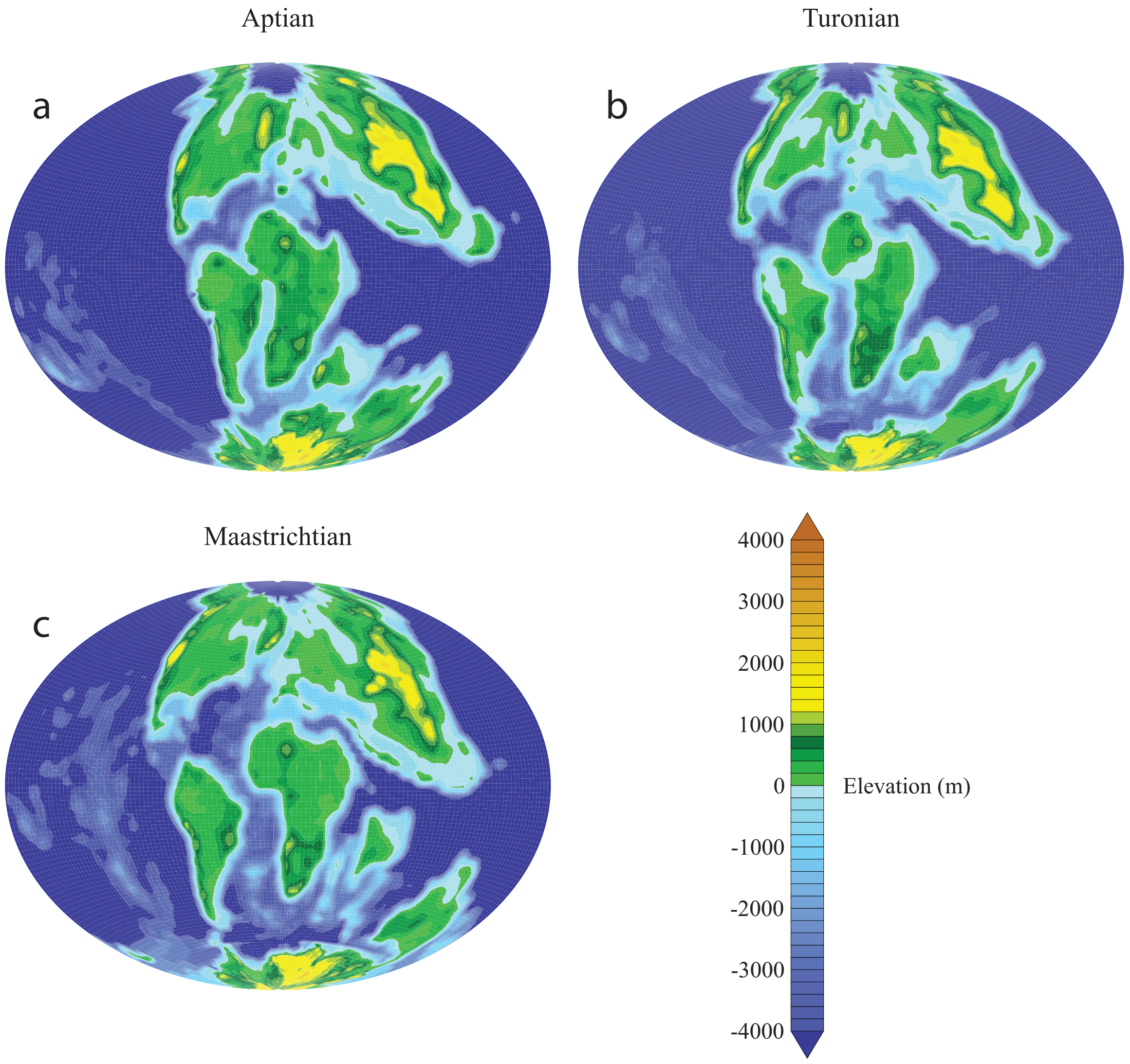

Supplementary Figure 1 - Aptian, Turonian and Maastrichtian palaeogeographies.

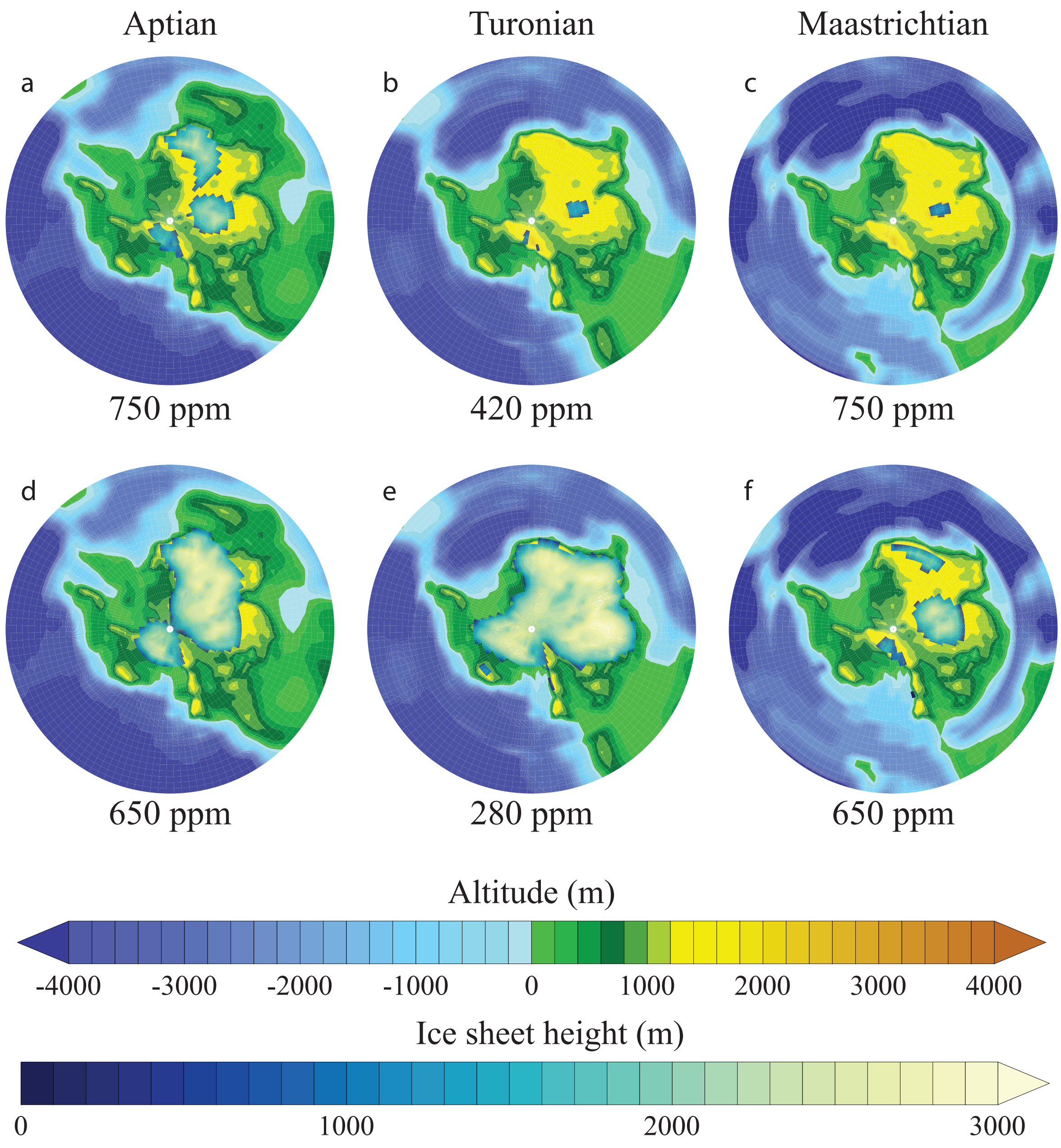

Supplementary Figure 2 - Simulated Antarctic ice sheet for additional CO_2 concentrations. The Ice Sheet Model is integrated for 10 kyrs under a constant cold austral summer orbit. Prescribed additional CO_2 concentrations are 750 and 650 ppm for the Aptian (a, d) and the Maastrichtian (c, f) and 420 and 280 ppm for the Turonian (b, e).

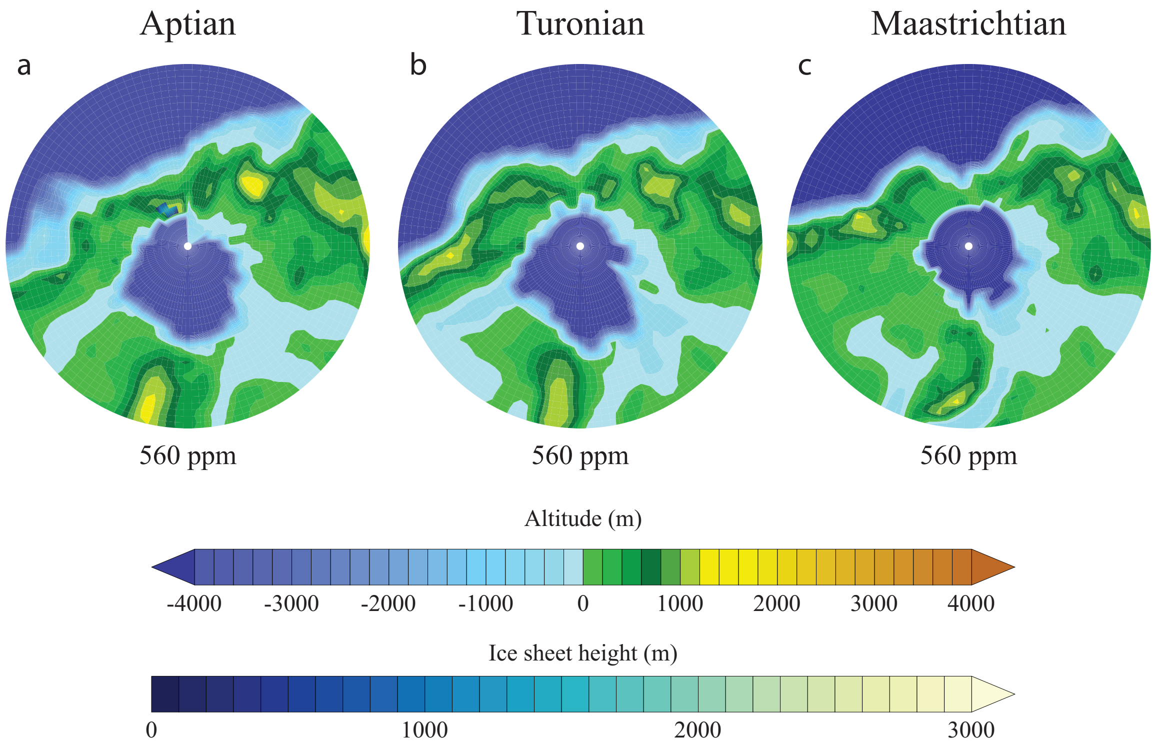

Supplementary Figure 3 - Simulated Northern Hemisphere ice sheet for each palaeogeography and a 560 ppm CO_2 concentration. The Ice Sheet model is integrated for 10 kyrs under a constant cold boreal summer orbit.

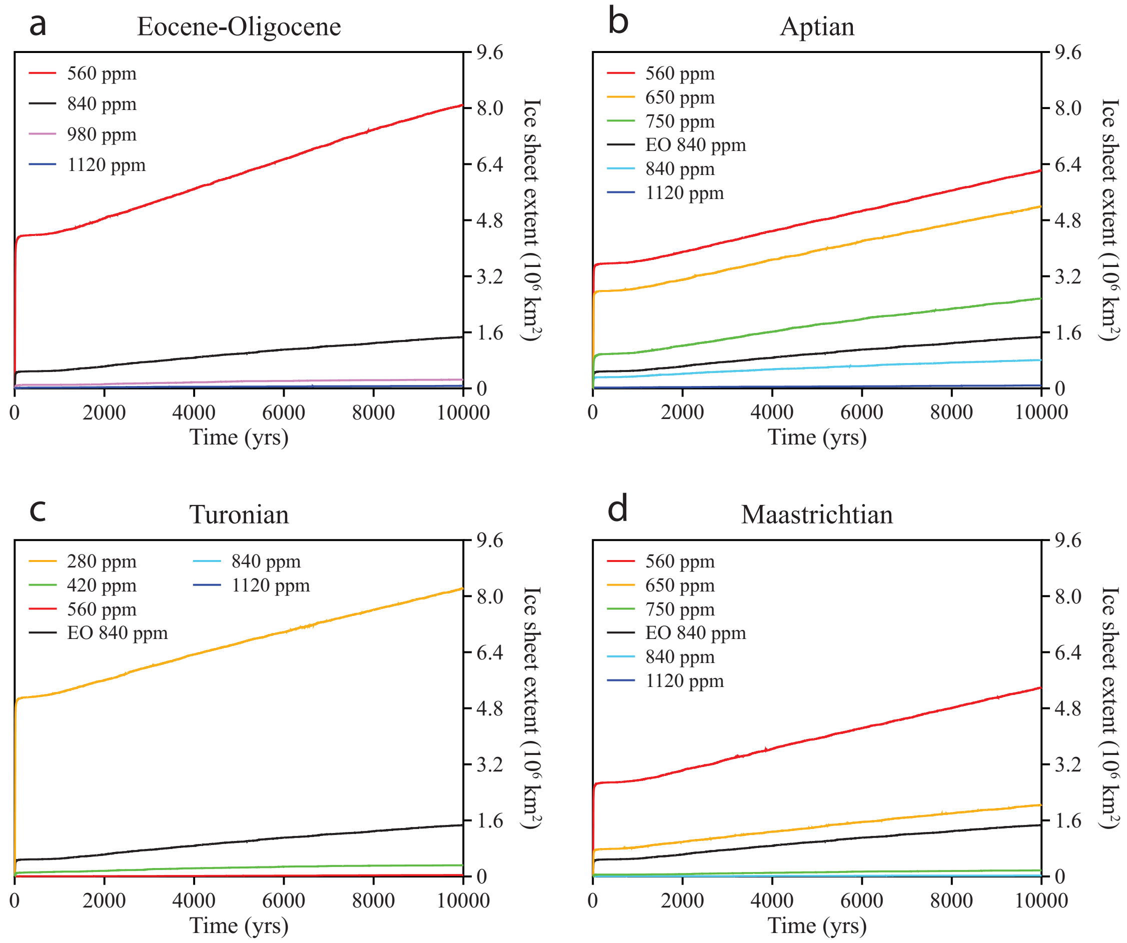

Supplementary Figure 4 – Ice sheet volume versus time for each simulation. (a) Eocene-Oligocene (EO) Antarctic ice sheet extent versus time obtained with the simple method at 560 ppm (red), 840 ppm (black), 980 ppm (purple) and 1120 ppm (blue). (b) Aptian Antarctic ice sheet extent at 560 ppm (red), 650 ppm (orange), 750 ppm (green), 840 ppm (light blue) and 1120 ppm (blue), and EO ice sheet extent at 840 ppm (black). (c) Same for the Turonian except that orange and green lines represent 280 and 420 ppm simulations respectively. (d) Same as (b) for the Maastrichtian. For the sake of simplicity, the simulated ice sheets obtained with the Cretaceous configurations are considered perennial when the simulated ice sheet extent after 10 kyrs of ISM integration under constant cold austral summer orbit is larger than the extent of the 840 ppm EO ice sheet (black lines in the EO and Cretaceous ice sheet subplots). Note that after 10 kyrs the simulated ice sheets are not in full equilibrium with climate thus ice sheet size or volume estimates cannot be inferred from these simulations.

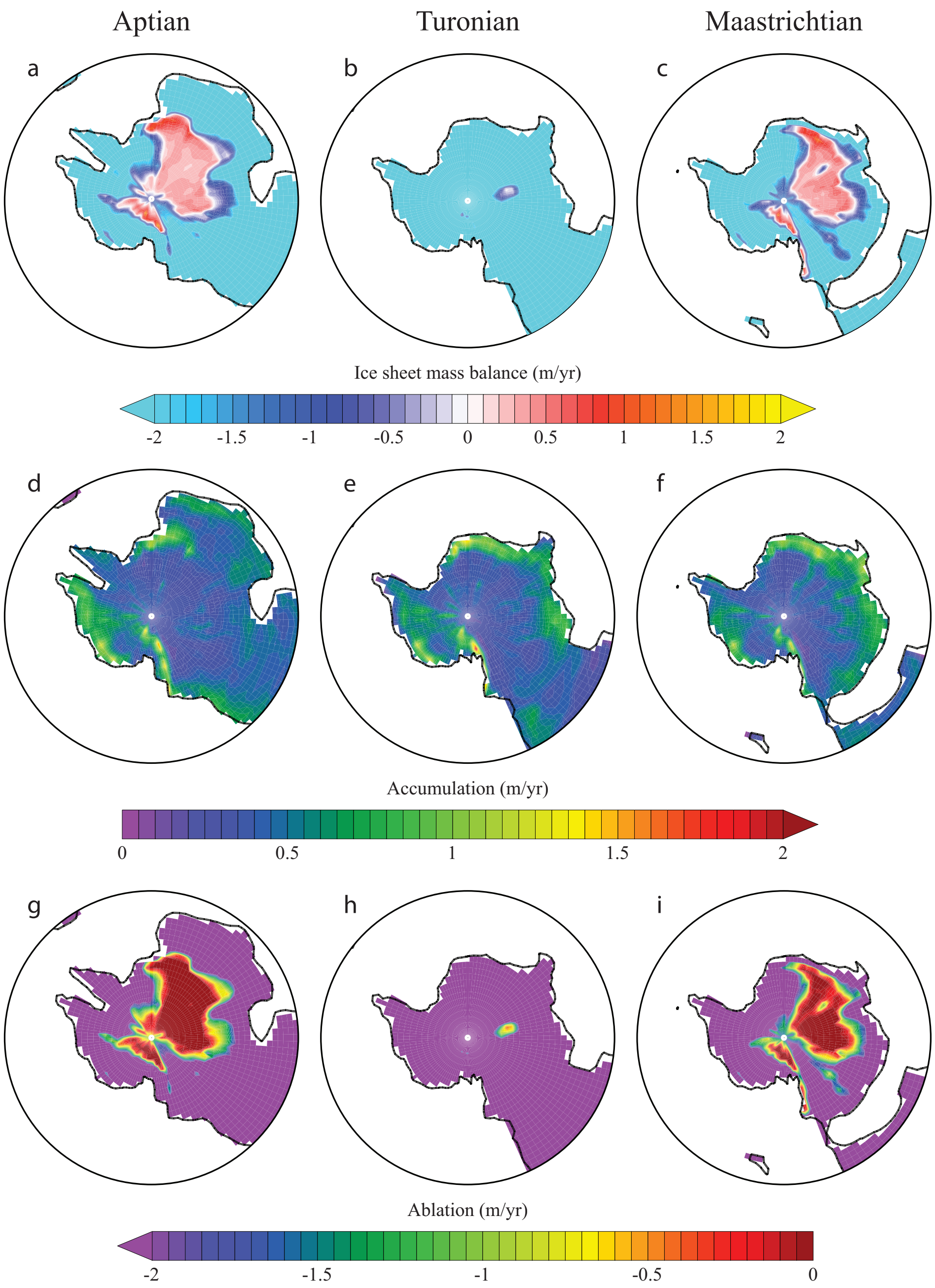

Supplementary Figure 5 – Initial ice sheet mass balance. Antarctic mass balance, accumulation and ablation terms for each palaeogeography at the beginning of the ice sheet simulations at 560 ppm.

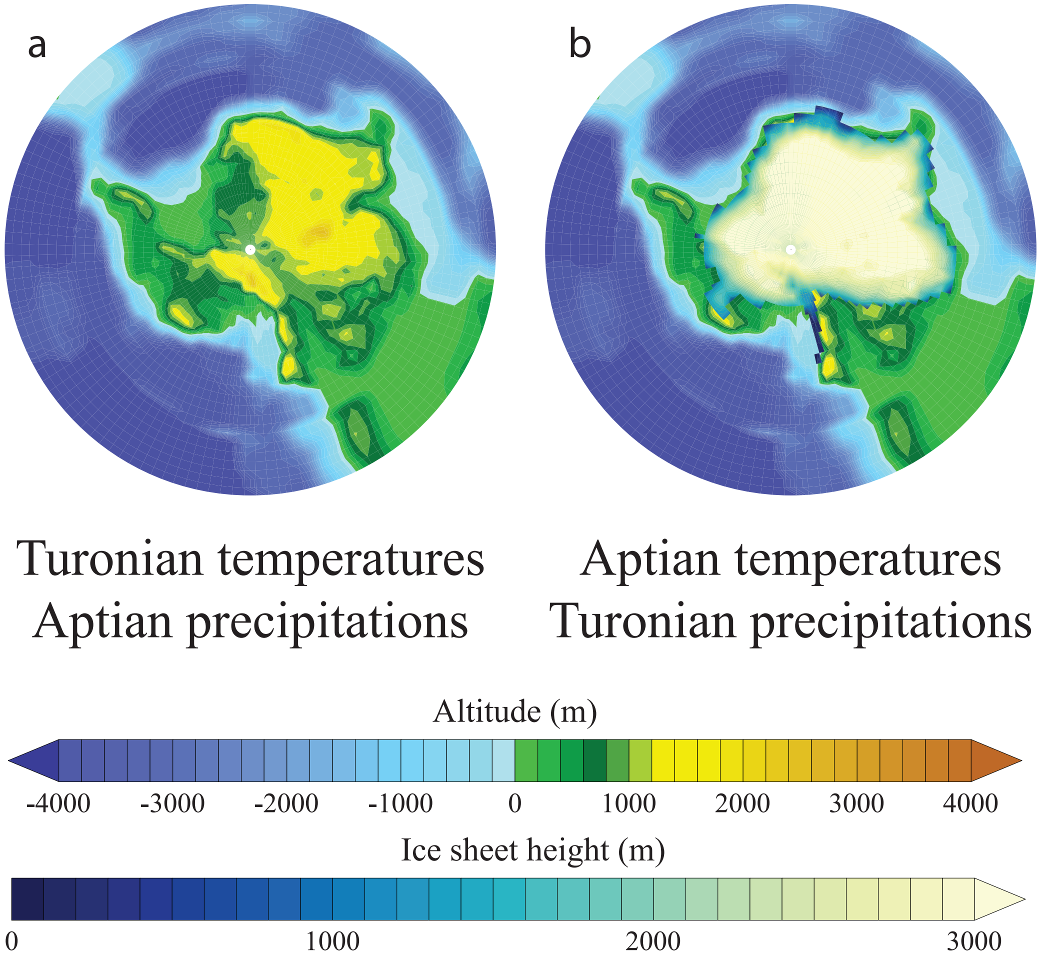

Supplementary Figure 6 – Turonian ice sheet sensitivity. Sensitivity tests to temperature and precipitation forcing fields of the Turonian ice sheet at 560 ppm after 10 kyrs of simulation. (a) Turonian temperature field and Aptian precipitation field. (b) Aptian temperature field and Turonian precipitation field.

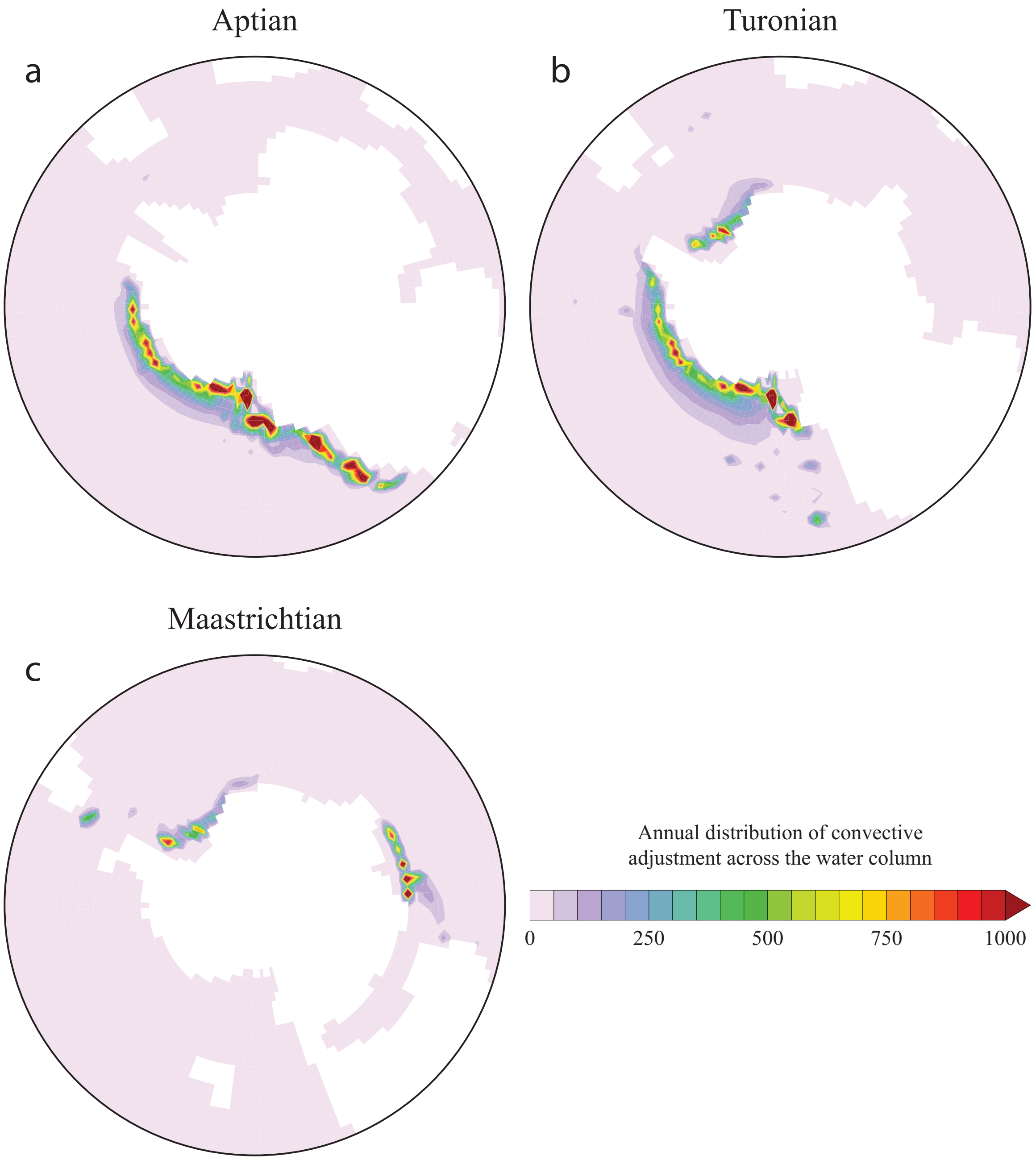

Supplementary Figure 7 – Annual oceanic convective adjustment. (a) Aptian, (b) Turonian and (c) Maastrichtian annual distribution of convective adjustment across the water column at 560 ppm. Areas with large convective activity are interpreted to represent intermediate to deep-water formation zones.

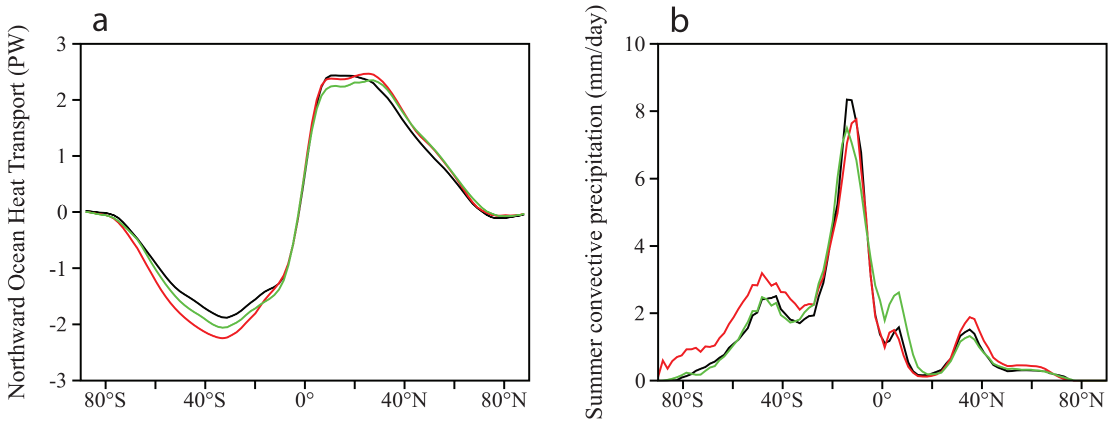

Supplementary Figure 8 - Northward Ocean Heat Transport (OHT) and zonally averaged summer convective precipitations. (a) Northward OHT for the Aptian (black), Turonian (red) and Maastrichtian (green) simulations at 560 ppm. (b) Summer convective precipitations for the Aptian (black), Turonian (red) and Maastrichtian (green) simulations at 560 ppm.

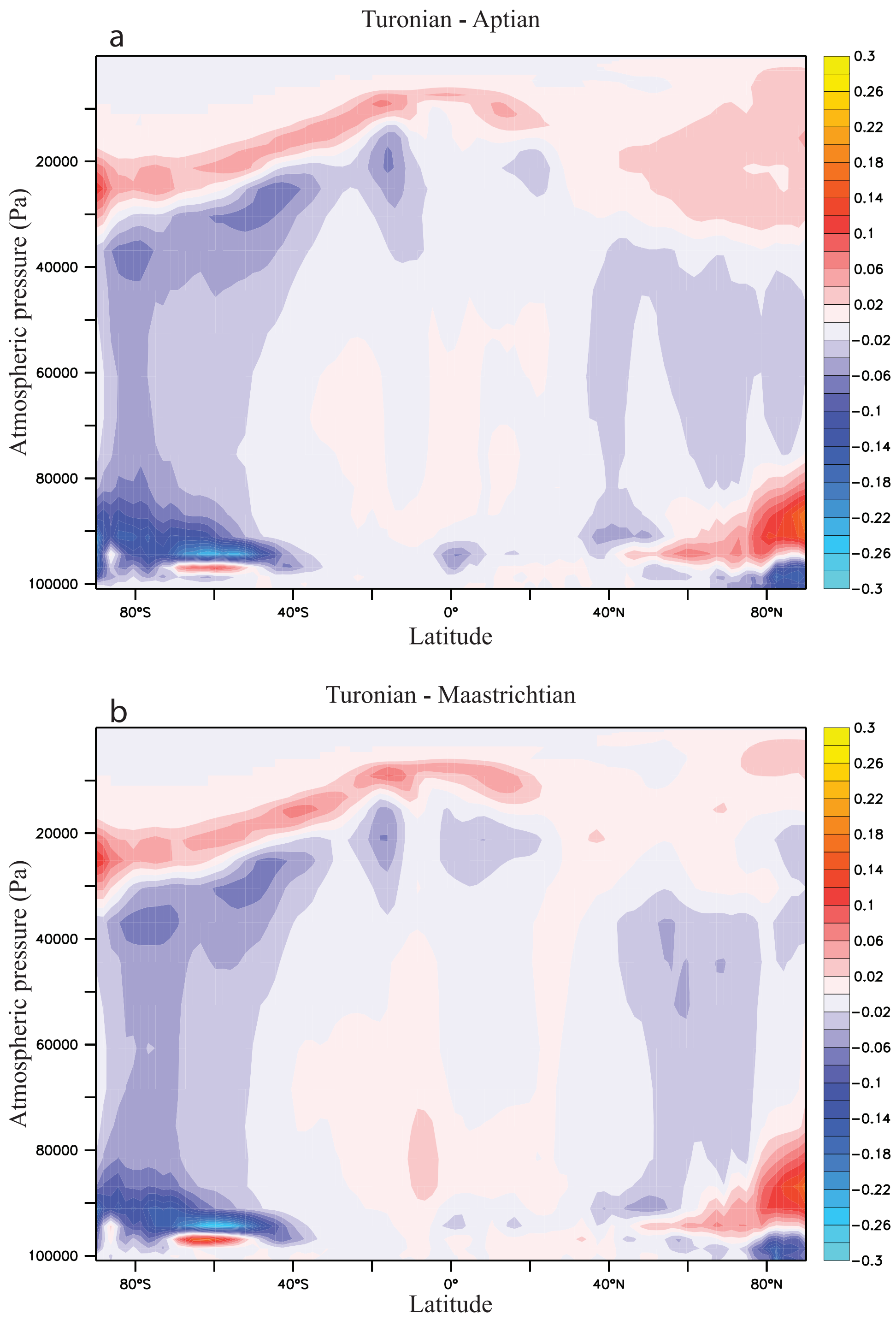

Supplementary Figure 9 – Cloud changes. Cloud fraction changes in the Turonian simulation with respect to the (a) Aptian and (b) Maastrichtian simulations at 560 ppm.

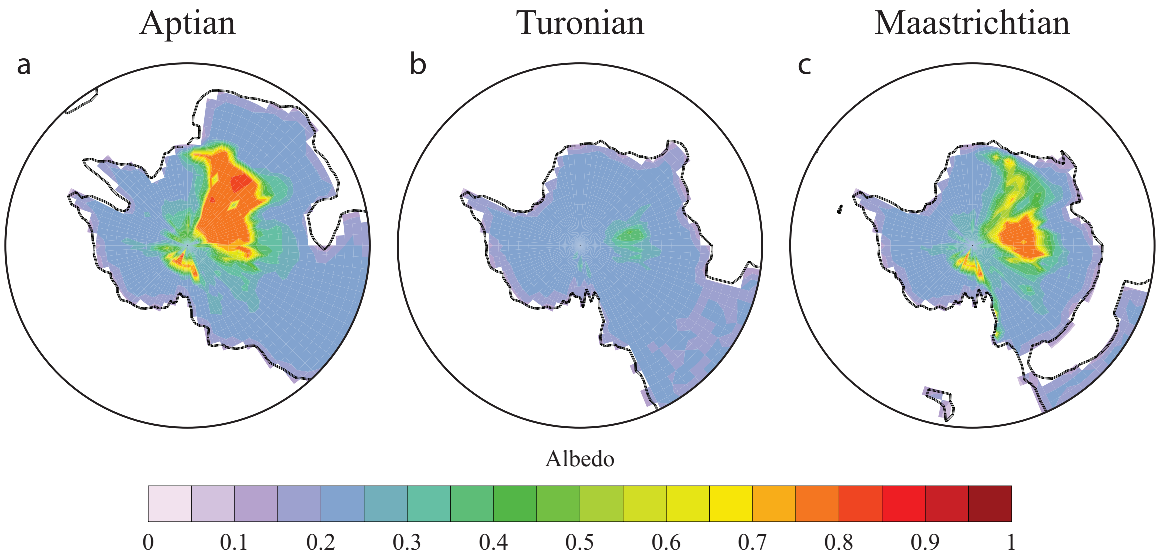

Supplementary Figure 10 – Summer albedo. Mean summer albedo for each paleogeography at 560 ppm.

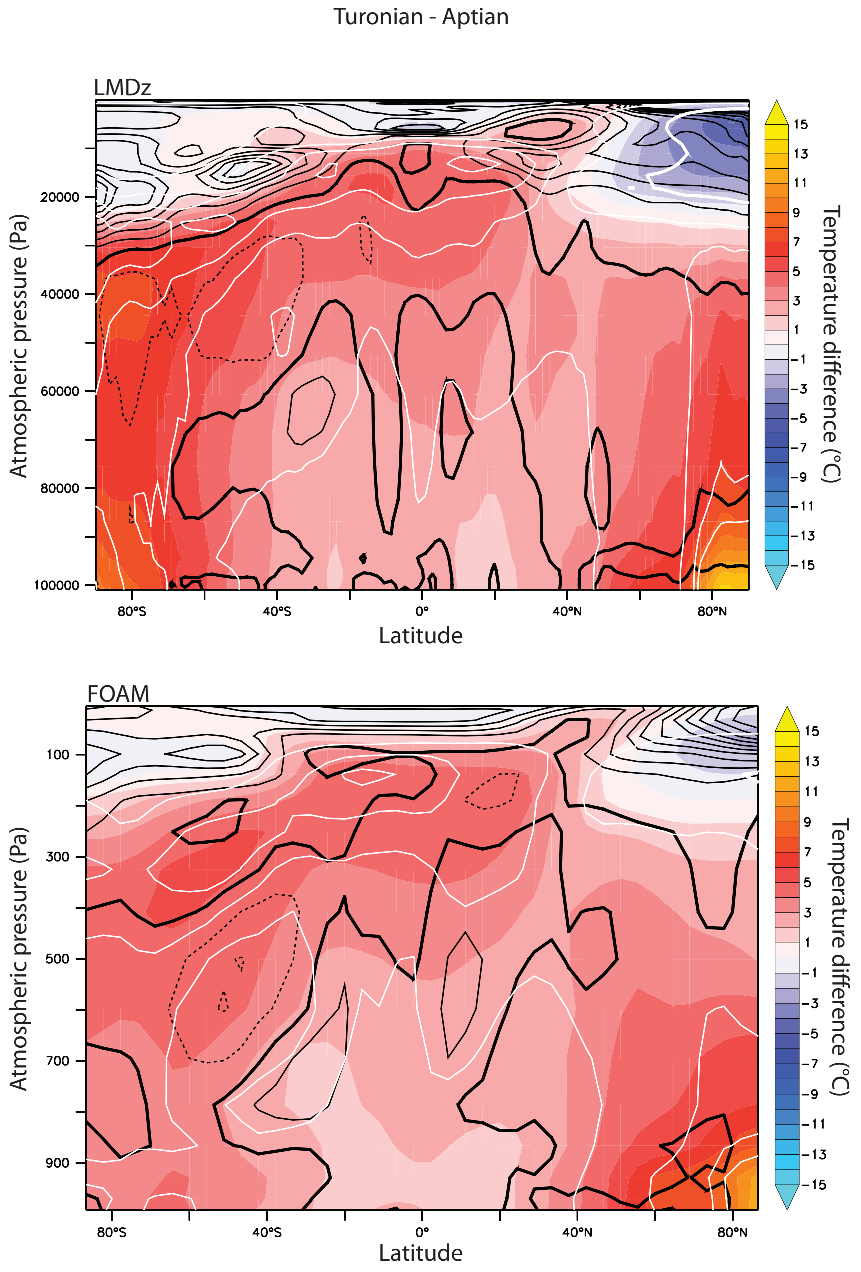

Supplementary Figure 11 – Turonian-Aptian temperature difference in LMDz and FOAM. Turonian minus Aptian vertical summer temperature difference at 560 ppm. White (black) contours are the Turonian minus Aptian changes in specific (relative) humidity. Top figure is the same as Fig. 3a.

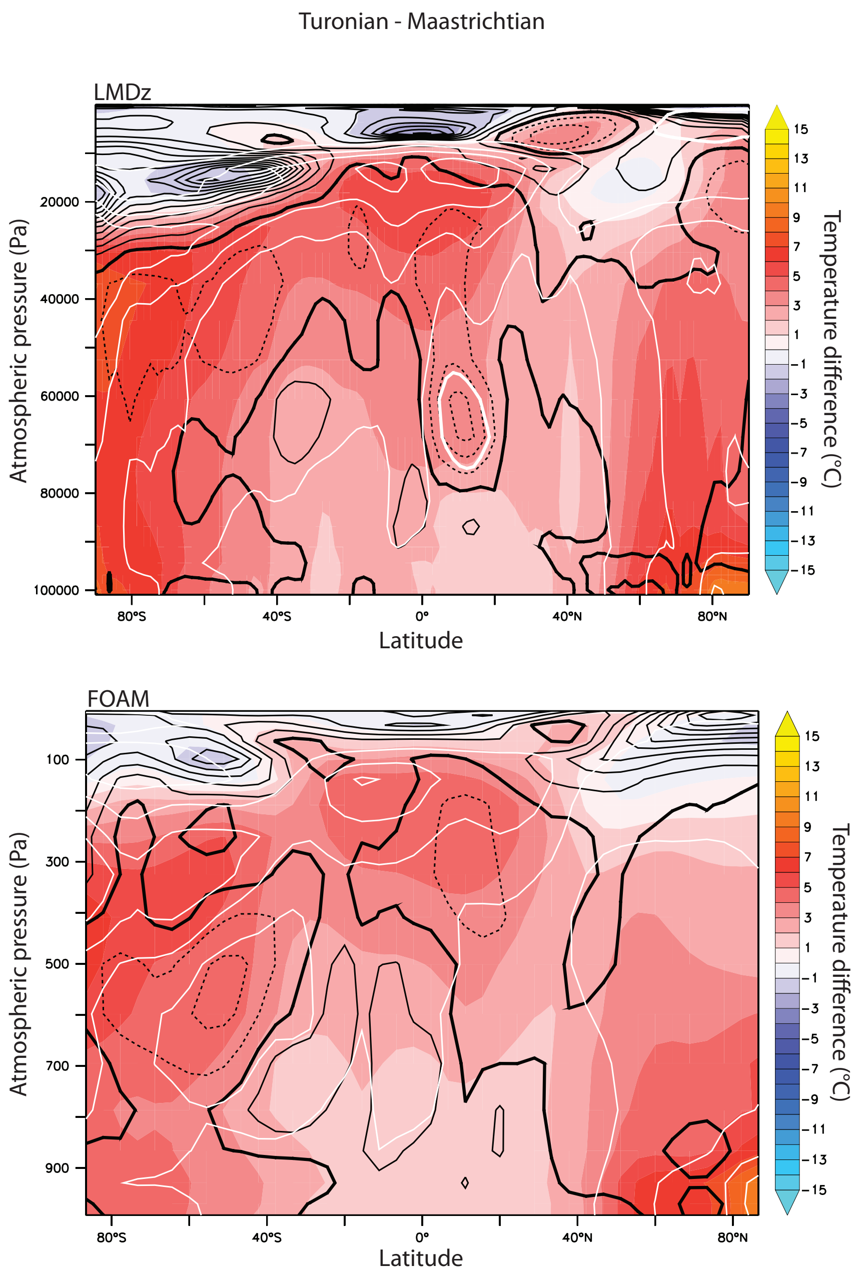

Supplementary Figure 12. Same as Supplementary Fig. 11 for the Turonian minus Maastrichtian changes. Top figure is the same as Fig. 3b.

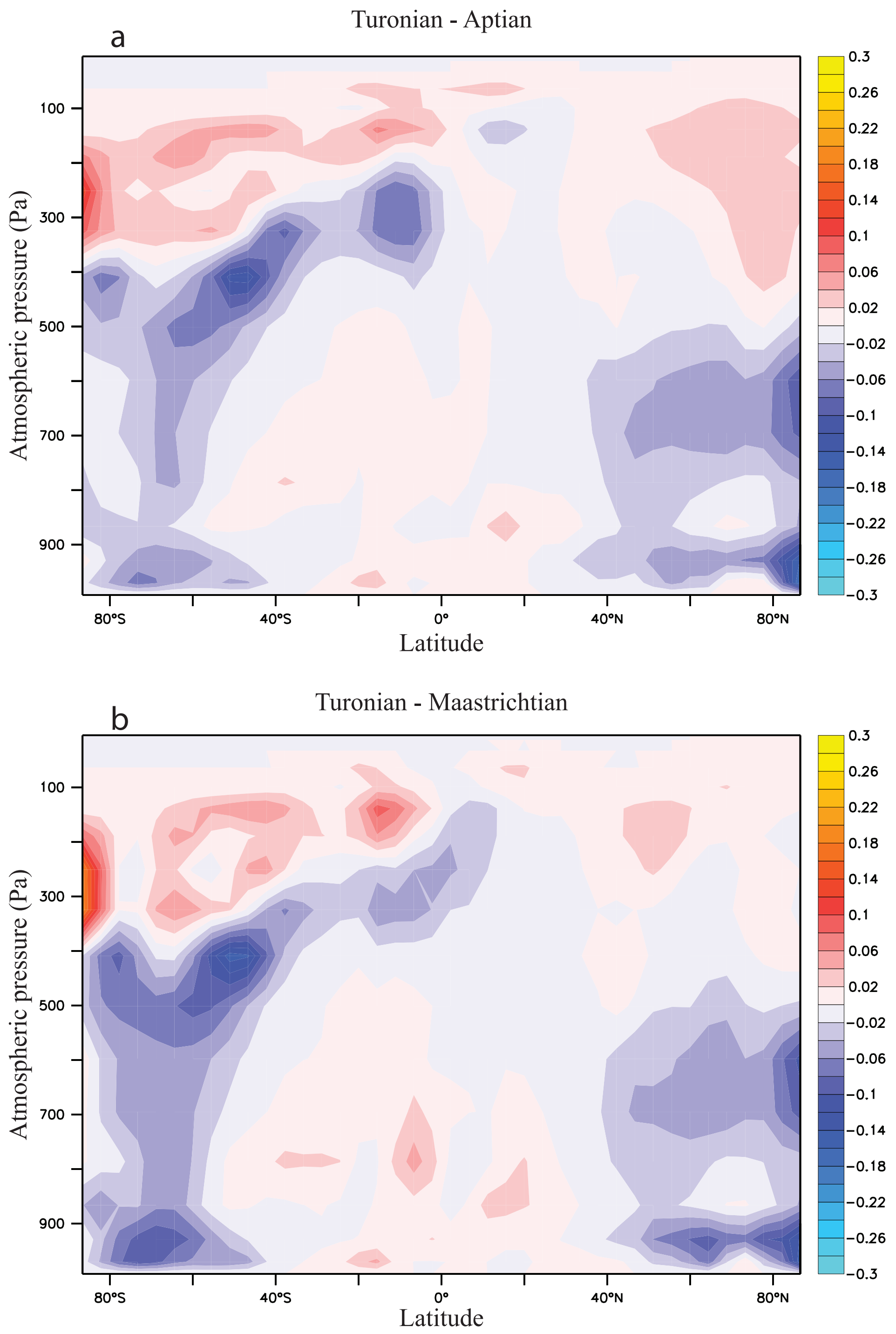

Supplementary Figure 13 – Cloud changes in FOAM. Cloud fraction changes in the Turonian with respect to the (a) Aptian and (b) Maastrichtian at 560 ppm in FOAM.

	CO_2 (ppm)	MAT (°C)	$Q_{500\ hPa}$ (g/kg)
	560	18.5	1.531
	650	19.1	1.618
Aptian	750	19.7	1.698
	840	20.0	1.753
	1120	21.3	2.003
	280	18.4	1.434
	420	20.6	1.748
	560	21.6	1.923
Turonian	650	22.1	2.023
	750	23.0	2.184
	840	23.4	2.288
	1120	24.4	2.545
	560	19.4	1.613
	650	19.9	1.701
Maastrichtian	750	21.0	1.876
	840	21.5	1.952
	1120	22.7	2.189

Supplementary Table 1 - Temperature and specific humidity for each palaeogeography and CO_2 levels. Globally averaged annual surface temperature (at 2 m) and specific humidity at 500 hPa for every combination of palaeogeography and atmospheric CO_2 levels.
